# Supplementary material for: Global neuropathologic severity of Alzheimer’s disease and locus coeruleus vulnerability influences plasma phosphorylated tau levels
Source: Mol Neurodegener. 2022 Dec 27;17:85. doi: 10.1186/s13024-022-00578-0 (PMC9795667; doi:10.1186/s13024-022-00578-0)
Supplement: Supplementary file 1 — Additional file 1. [file 13024_2022_578_MOESM1_ESM.docx]

**Supplementary Appendix**

| **Section** | **Page** |
| --- | --- |
| **Supplemental Methods** | 2 |
| Plasma p-tau assays | 2 |
| Digital pathology procedures | 2 |
| Tau PET procedures | 2 |
| **Supplemental Results** | 3 |
| Clinical diagnosis across autopsy cohort | 3 |
| Antemortem contributors to plasma p-tau levels | 3 |
| Tau PET signal in parietal cortex associates with plasma p-tau levels | 3 |
| Alzheimer’s disease neuropathologic change predicted better by cognitive measures than plasma p-tau | 3 |
| Table S1. Antibody table | 4 |
| Table S2. Digital pathology color deconvolution macros for pT181 and pT217. | 5 |
| Table S3. Positive pixel count macro specifications for 6F/3D. | 6 |
| Table S4. Nuclear macros for neuron count in nucleus basalis of Meynert and locus coeruleus. | 7 |
| Table S5. Multivariable regression analysis of neuropathologic predictors of plasma p-tau. | 8 |
| Table S6: Multivariable linear regression models predicting cognitive scores. | 9 |
| Table S7. Clinical diagnosis and availability of tau PET within each neuropathologic grouping. | 10 |
| Figure S1. Immunohistochemical staining from hippocampus across disease groups. | 11 |
| Figure S2. The rostral-to-caudal extent of the locus coeruleus. | 12 |
| Figure S3. The anterior-to-posterior extent of the nucleus basalis of Meynert. | 13 |
| Figure S4. Digital pathology analysis of nucleus basalis of Meynert and locus coeruleus. | 14 |
| Figure S5. Evaluation of antemortem contributors to plasma p-tau variability. | 15 |
| Figure S6. Regional digital pathology measures of tau and amyloid-β pathology in hippocampus. | 16 |
| Figure S7. Digital pathology measures of nucleus basalis of Meynert and locus coeruleus. | 17 |
| Figure S8. Evaluation of [^18^F]flortaucipir uptake in inferior parietal cortex and plasma p-tau levels. | 18 |
| Figure S9. Predictive modeling of Alzheimer’s disease neuropathologic change. | 19 |
|  |  |
| **Supplemental References** | 20 |

# Supplemental Methods

## Plasma p-tau assays

Phosphorylated tau at threonine181 (p-tau181) and threonine217 (p-tau217) levels were measured in duplicate on a streptavidin small spot plate using the meso scale discovery (MSD) platform by electrochemiluminescence using proprietary assays developed by Lilly Research Laboratories, as previously described [1]. Briefly, samples were diluted 1:2 and 50 uL of diluted sample was used for each replicate. P-tau181 used biotinylated-AT270 (mouse IgG1) as the capture and p-tau217 used biotinylated-IBA493 (mouse IgG1) as the capture. In this study, both assays used SULFO-4G10-E2 (anti-tau monoclonal antibody developed by Lilly Research Laboratories) as the detector. Each assay was calibrated using a unique synthetic p-tau peptide coupled with a polyethylene glycol linker to a second tau peptide matching amino acid 111-130 according to the Tau441 sequence numbering.

## Digital pathology procedures

Hematoxylin and eosin (H&E), as well as immunohistochemically stained slides, were digitally scanned at 20x using the Aperio AT2 scanner and annotated using ImageScope (Leica Biosystems, version 12.4.3.5008). The CA1 and subiculum subsectors of the hippocampus were traced on serial sections as previously described [2]. Briefly, the superior border was defined as the boundary between the lacunosum and radiatum layers. The inferior border was defined as the boundary between the pyramidal layer and alveus. To operationalize using neuroanatomic landmarks, the CA1 and subiculum were collectively traced from the ventricle following the alveus medially to the rise of the presubiculum. The inferior parietal cortex was traced using the pial surface and gray-white junction as borders along the extent of the gray matter. Given the lack of discrete boundaries in the locus coeruleus, a 1.8x1.8 mm^2^ box was placed centering the locus coeruleus neurons based upon maximization of locus coeruleus neurons encompassed in 10 healthy control brains. The level (i.e. rostral-to-caudal extent) of the locus coeruleus was assessed using appearance of the superior cerebellar peduncle, velum, cerebral aqueduct, and fourth ventricle (**Fig. S2**) [3]. The middle locus coeruleus level was selected for analysis based on prior studies demonstrating topographic vulnerability of rostral and middle level to AD [3, 4] and wider availability in the study cohort (n=28/35 [80%]). The nucleus basalis of Meynert was annotated as previously described [5]. Briefly, the lateral edge of the nucleus basalis of Meynert was defined by where the globus pallidus and putamen meet perpendicular to the ventral surface of the brain. The medial edge was defined by a perpendicular line from the lateral end of the optic nerve up to the globus pallidus, or the most medial part of the internal capsule if the optic nerve was not present. The anterior commissure was dorsal to the annotated region. The nucleus basalis of Meynert level (i.e., anterior-to-posterior extent) was assigned using the location and appearance of the anterior commissure, globus pallidus, fornix, mammillary body (**Fig. S3**). The anterior nucleus basalis of Meynert level was selected for analysis because this level contains the most widespread and noticeable portion of the nucleus basalis of Meynert [5-7], along with wider availability in the study cohort (n=24/35 [69%]).

GENIE, a pattern recognition software (Leica Biosystems, v12.4.3.7001) was used to better automate locus coeruleus and nucleus basalis of Meynert neuron counts (**Fig. S4**). Annotated examples of each classifier were used to train until a mean training accuracy >85% was reached with minimal errors identified visually. For the locus coeruleus, four classifiers were created: locus coeruleus neurons, blood vessels, neuropil, and white matter. After 1,000 iterations of training, a mean training accuracy of 98.28% was reached. The classifiers were then incorporated into a nuclear macro so that regions classified as “LC neurons” and “neuropil” would be analyzed. For the nucleus basalis of Meynert, five classifiers were created: nucleus basalis of Meynert neurons, blood vessels, neuropil, corpora amylacea, and small vessels. After 2,000 iterations of training, a mean training accuracy of 88.19% was reached. The classifiers were then incorporated into a nuclear macro so that regions classified as “nbM neurons” and “neuropil” would be analyzed.

eSlideManager (Leica Biosystems) was used to analyze digital images of the hippocampus and parietal cortex. Slides were batch analyzed using customized color deconvolution and positive pixel count macros designed for each antibody to recognize the 3, 3'-diaminobenzidine staining on the tissue and to exclude background to obtain burden (**Tables S2-S3**). Scans of the locus coeruleus and nucleus basalis of Meynert batch analyzed using customized nuclear macros designed for each region, including their GENIE classifiers (**Table S4**).

## Tau PET procedures

To contextualize the strength of the relationship between regional tau measures and plasma p-tau levels, antemortem tau positron emission tomography (PET) uptake was quantified in inferior parietal cortex for 10 cases with available data (**Table S7**). Tau PET measures from hippocampus are often difficult to ascertain and were thus not investigated. [^18^F]Flortaucipir tau PET images were acquired using a PET/CT scanner operating in 3-dimensional mode, as previously described in detail [8]. Inferior parietal cortex region of interest (ROI) was defined by an in-house version of the automated anatomic labelling atlas as previously described. The tau PET ROI median inferior parietal cortex values were normalized to cerebellar crus (bilateral crus, 1-2) to calculate regional standardized uptake value ratios (SUVr) [8].

# Supplemental Results

## Clinical diagnosis across autopsy cohort

Autopsied participants included cognitively unimpaired (n=25 [71%]), mild cognitive impairment (n=4 [11%]), AD dementia (n=3 [9%]), and non-AD neurodegenerative disorders (n=3 [9%]) (**Table S7**).

## Antemortem contributors to plasma p-tau levels

Because previous studies suggested that comorbidities, including both kidney and liver disease, may affect p-tau biomarker levels,[9, 10] we first examined Spearman correlations of creatinine, aspartate aminotransferase (AST), and alanine aminotransferase (ALT) with plasma p-tau levels. Graphs of creatinine and plasma p-tau levels are shown in **Figure S5-A, F**. Serum creatinine levels correlated with plasma p-tau181 (R=0.34, p=0.049) and approached significance with plasma p-tau217 (R=0.31, p=0.074). One individual was an outlier with high serum creatinine (3.7 mg/dL). This person also had the highest plasma p-tau181 (10 pg/mL) and plasma p-tau217 (1.3 pg/mL) levels of the cohort. Neuropathologic investigation of this 90-year-old man’s brain revealed argyrophilic grains disease and pathological aging (Braak III, Thal 3), which was deemed insufficient to account for the high plasma p-tau levels. Exclusion of the outlier diminished the relationship between creatinine and p-tau181 (R=0.27, p=0.116), as well as plasma p-tau217 (R=0.24, p=0.169). This individual remained part of the initial analyses evaluating antemortem variability of creatinine, AST, ALT, age at plasma p-tau draw, and time from blood draw to death, but was removed from subsequent analyses investigating cognition and neuropathology. We next examined liver health with a focus on serum AST (**Figure S5-B, G**) and ALT liver enzyme levels (**Figure S5-C, H**). Serum AST levels did not correlate with plasma p-tau181 (R=-0.21, p=0.257) or with plasma p-tau217 (R=-0.12, p=0.526). Similarly, we did not observe a correlation between serum ALT levels and plasma p-tau181 (R=0.14, p=0.553) or with plasma p-tau217 (R=0.14, p=0.564). The 90-year-old male participant with high serum creatinine levels (3.7 mg/dL) had the shortest time from plasma draw to death (0.3 years). Exclusion of this participant did not improve the relationship between either age at plasma draw or time from plasma draw to death with plasma p-tau levels.

## Tau PET signal in parietal cortex associates with plasma p-tau levels

A subset of 10 individuals with antemortem [^18^F]flortaucipir PET were examined for their relationship between radioligand uptake in parietal cortex and plasma p-tau levels to provide in vivo comparison (**Table S7**, **Fig. S8**). The magnitude of the correlation between parietal cortex tau PET uptake and plasma p-tau181 was high, but did not reach statistical significance (R=0.54, p=0.113). We observed an even stronger relationship between parietal cortex tau PET uptake and plasma p-tau217 (R=0.71, p=0.028).

## Alzheimer’s disease neuropathologic change predicted better by cognitive measures than plasma p-tau

Although we hypothesized that accumulation of neuropathology influences plasma p-tau levels, we evaluated receiver operating characteristics (ROC) curves with corresponding area under the curves (AUC) to assess the utility of plasma p-tau and cognitive scores to independently prognosticate neuropathologic classification of intermediate-to-high from none-to-low AD neuropathologic change (**Fig. S9**). Given the sample size and use of logistic regression, plasma p-tau levels and cognitive scores were modeled individually without covariates. P-tau217 had the highest predictive value for AD neuropathologic change (AUC=0.89 [CI=0.75, 1.0]), followed by CDR sum of boxes (AUC=0.81 [0.66, 0.95]), p-tau181 (AUC=0.80 [CI=0.62, 0.95]), and MMSE (AUC=0.76 [CI=0.56, 0.93]).

# Supplemental Tables

**Table S1. Antibody table.**

| **Antibody Name** | **Clone** | **Conc** | **Host** | **Source** | **Catalog #** | **Epitopes** |
| --- | --- | --- | --- | --- | --- | --- |
| **Murray et al Diagnostic antibodies** | | | | | | |
| Tau (AT8) | Monoclonal (AT8) | 1:100 | Ms | ThermoFisher | MN1020 | pS202/pT205 |
| Amyloid-β | Monoclonal (6F/3D) | 1:100 | Ms | DAKO | M0872 | 8-17 Aβ with additional C-terminal cysteine coupled to keyhole limpet hemocyanin [KLH] via the cysteine residue |
| Phospho-TDP-43 | Monoclonal (pS409/410) | 1:10,000 | Ms | Cosmo Bio | TIP-PTD-M01 | pS409/pS410 |
| α-Synuclein | Monoclonal (LB509) | 1:25 | Ms | ThermoFisher | 180215 | α-synuclein amino acids 115-122 |
| **Murray et al Digital pathology antibodies** | | | | | | |
| Tau (pT181) | Monoclonal (pT181) | 1:10,000 | Ms | ThermoFisher | MN1050 | pT181 |
| Tau (pT217) | Polyclonal (pT217) | 1:500 | Rb | ThermoFisher | 44-744 | pT217 |
| Amyloid-β | Monoclonal (6F/3D) | 1:100 | Ms | DAKO | M0872 | 8-17 Aβ with additional C-terminal cysteine coupled to keyhole limpet hemocyanin [KLH] via the cysteine residue |
| **Murray et al Meso Scale Discovery capture antibodies** | | | | | | |
| Biotinylated-AT270 (p-tau181) | Monoclonal (pT181) | 1 µg/mL | Ms | ThermoFisher | MN1050 | pT181 |
| Biotinylated-IBA493 (p-tau217) | Monoclonal (pT217) | 0.5 µg/mL | --- | Eli Lilly | --- | pT217 |
| **Murray et al Meso Scale Discovery detector antibody** | | | | | | |
| SULFO-4G10-E2 (Tau) | Monoclonal | --- | --- | Eli Lilly | --- | --- |
| **Wennström et al Immunostaining antibodies** | | | | | | |
| Tau (AT270) | Monoclonal (pT181) | --- | Ms | ThermoFisher | MN1050 | pT181 |
| Tau (AT8) | Monoclonal (AT8) | --- | Ms | ThermoFisher | MN1020 | pS202/pT205 |
| Tau (CP13) | Monoclonal (pS202) | --- | Ms | Dr. Peter Davies | --- | pS202 |
| Tau (IBA413) | --- | --- | Rb | Eli Lilly | --- | --- |
| Tau (EPR2488) | Monoclonal (pT231) | --- | Rb | Abcam | ab151559 | pT231 |
| Tau (PHF1) | Monoclonal (pS396/pS404) | --- | Ms | Dr. Peter Davies | --- | pS396/pS404 |
| CD63 (MEM-259) | Monoclonal (MEM-259) | --- | Ms | ThermoFisher | MA1-19281 | T cell line HPB-ALL |
| Ckid (128a) | --- | --- | Ms | Eli Lilly | --- | --- |
| **Wennström et al Meso Scale Discovery capture antibody** | | | | | | |
| Biotinylated-IBA493 (p-tau217) | --- (pT217) | --- | Rb | Eli Lilly | --- | pT217 |
| **Wennström et al Meso Scale Discovery detector antibody** | | | | | | |
| SULFO-4G10-E2 (Tau) | Monoclonal | --- | --- | Eli Lilly | --- | --- |

--- indicates missing information or not applicable. Conc=concentration. Ms=Mouse. Rb=Rabbit

**Table S2. Digital pathology color deconvolution count macros for pT181 and pT217.**

| **Version** | v9 |
| --- | --- |
| **View Width** | 1000 |
| **View Height** | 1000 |
| **Overlap Size** | 0 |
| **Image Zoom** | 1 |
| **Markup Compression Type** | Same as processed image |
| **Compression Quality** | 30 |
| **Classifier Neighborhood** | 0 |
| **Classifier** | None |
| **Class List** |  |
| **Positive Color Channel** | 3 |
| **Mark-up Image Type** | Intensity Ranges |
| **Weak Positive Threshold** | 235 |
| **Medium Positive Threshold** | 180 |
| **Strong Positive Threshold** | 180 |
| **Black Threshold** | 0 |
| **Color (1) - Red Component** | 0.65 |
| **Color (1) - Green Component** | 0.704 |
| **Color (1) - Blue Component** | 0.286 |
| **Color (2) - Red Component** | 0.072 |
| **Color (2) - Green Component** | 0.99 |
| **Color (2) - Blue Component** | 0.105 |
| **Color (3) - Red Component** | 0.341 |
| **Color (3) - Green Component** | 0.575 |
| **Color (3) - Blue Component** | 0.743 |
| **Clear Area Intensity** | 240 |
| **Display Plots** | No |

**Table S3. Positive pixel count macro specifications for 6F/3D.**

| **Version** | **v9** |
| --- | --- |
| **View Width** | 1000 |
| **View Height** | 1000 |
| **Overlap Size** | 0 |
| **Image Zoom** | 1 |
| **Markup Compression Type** | Same as processed image |
| **Compression Quality** | 30 |
| **Classifier Neighborhood** | 0 |
| **Classifier** | None |
| **Class List** |  |
| **Hue Value** | 0.1 |
| **Hue Width** | 0.1 |
| **Color Saturation Threshold** | 0.08 |
| **Iwp (High)** | 235 |
| **Iwp(Low) = Ip(High)** | 235 |
| **Ip(Low) = Isp(High)** | 235 |
| **Isp(Low)** | 0 |
| **Inp(High)** | -1 |

**Table S4. Nuclear macros for digital pathology.**

|  | | **Locus coeruleus** | **Nucleus basalis of Meynert** |
| --- | --- | --- | --- |
| **Version** | | v9 | v9 |
| **Stain 1** | S1 Number of Visible Stains | 2 | 2 |
|  | S1 Target | Counterstain | Counterstain |
|  | S1 Color | Lock | Lock |
|  | S1 Values | Hide | Hide |
|  | --Stain-1 (Red) | 0.268 | 0.268 |
|  | --Stain-1 (Green) | 0.570 | 0.570 |
|  | --Stain-1 (Blue) | 0.776 | 0.776 |
| **Stain 2** | S2 Number of Visible Stains | 2 | 2 |
|  | S2 Target | Biomarker 1 | Biomarker 1 |
|  | S2 Color | Lock | Lock |
|  | S2 Values | Hide | Hide |
|  | --Stain-2 (Red) | 0.268 | 0.268 |
|  | --Stain-2 (Green) | 0.570 | 0.570 |
|  | --Stain-2 (Blue) | 0.776 | 0.776 |
| **Stain 3** | S3 Number of Visible Stains | 0 | 0 |
|  | S3 Target |  |  |
|  | S3 Color |  |  |
|  | S3 Values |  |  |
|  | --Stain-3 (Red) |  |  |
|  | --Stain-3 (Green) |  |  |
|  | --Stain-3 (Blue) |  |  |
| **Nuclei Identification** | Type | All Stains | All Stains |
|  | Method | Average | Average |
|  | --Threshold Lower Limit | 0 | 0 |
|  | --Threshold Upper Limit | 245 | 255 |
|  | Smoothing (µm) | 2.5 | 2.5 |
|  | Merging | 2 | 3 |
|  | Trimming | Low | Low |
| **Nuclei Exclusion** | Min Size (µm^2) | 200 | 200 |
|  | Max Size (µm^2) | 5000 | 5000 |
|  | Roundness | 0.1 | 0.01 |
|  | Compactness | 0.1 | 0.01 |
|  | Elongation | 0.05 | 0.05 |
|  | Remove Light Objects | 0 | 0 |
| **Scoring Criteria** | Cytoplasmic Correction | 255 | 255 |
|  | Weak(1+) Threshold | 255 | 255 |
|  | Moderate(2+) Threshold | 255 | 255 |
|  | Strong(3+) Threshold | 0 | 0 |
|  | Dark Nuclei Removal | 0 | 0 |
| **Plots** | Display Plots | No | No |
| **Advanced** | Image Zoom | 1 | 1 |
|  | Markup Compression Type | Same as processed image | Same as processed image |
|  | Compression Quality | 30 | 30 |
|  | Classifier Neighborhood | 50 | 98 |
|  | Class List | LC Neurons, Neuropil | nbM Neurons, Neuropil |
|  | Clear Area Intensity | 240 | 240 |

**Table S5:** **Multivariable linear regression models investigating contribution of co-existing pathology to the associations between global scales and regional neuropathologic measures to plasma p-tau levels**

| **Tau & Amyloid-β** | **Outcome** | **Adj R^2^** | **Predictor** | **β-coefficient** | **LowerCL** | **UpperCL** | **p-value** |
| --- | --- | --- | --- | --- | --- | --- | --- |
| **Global** | MSD p-tau181 | 0.31 | Braak stage | 0.2681 | -0.0726 | 0.6089 | 0.1184 |
|  |  |  | Thal phase | 0.3269 | 0.0528 | 0.6010 | 0.0211 |
|  |  |  | LATE-NC stage | 0.0051 | -0.5008 | 0.5109 | 0.9838 |
|  |  |  | Kalaria CVD score | 0.0111 | -0.1622 | 0.1844 | 0.8966 |
|  | MSD p-tau217 | 0.59 | Braak stage | 0.0601 | 0.0120 | 0.1081 | 0.0160 |
|  |  |  | Thal phase | 0.0803 | 0.0417 | 0.1190 | 0.0002 |
|  |  |  | LATE-NC stage | 0.0132 | -0.0581 | 0.0845 | 0.7085 |
|  |  |  | Kalaria CVD score | -0.0039 | -0.0284 | 0.0205 | 0.7442 |
| **Regional** | MSD p-tau181 | 0.24 | Parietal pT181 | 0.6620 | -0.5770 | 1.9010 | 0.2804 |
|  |  |  | Parietal 6F3D | 0.2678 | -0.0560 | 0.5916 | 0.1005 |
|  |  |  | LATE-NC stage | 0.1171 | -0.4697 | 0.7039 | 0.6835 |
|  |  |  | Kalaria CVD score | -0.0372 | -0.2637 | 0.1894 | 0.7374 |
|  | MSD p-tau217 | 0.51 | Parietal pT217 | 0.0217 | -0.0222 | 0.0656 | 0.3209 |
|  |  |  | Parietal 6F3D | 0.0766 | 0.0262 | 0.1270 | 0.0042 |
|  |  |  | LATE-NC stage | 0.0250 | -0.0526 | 0.1027 | 0.5151 |
|  |  |  | Kalaria CVD score | 0.0072 | -0.0183 | 0.0327 | 0.5683 |

Analyses were performed on n=34 individuals for p-tau217. Analyses were performed on n=28 individuals for p-tau181 as a result of immunohistochemical exclusion in 5 cases with heavy pT181 staining appearing in axons disproportionate to tau pathology. Case with high creatinine was not included. All variables in model are shown. Time from plasma draw to death was not used to adjust, as it was not observed to associate with plasma p-tau levels. Variance inflation factor for all terms were below 3, which fell below the general cutoff of 5 used to indicate multicollinearity. Acronyms: 6F/3D=amyloid-β antibody. Adj=adjusted, CL=95% confidence limit. Acronyms: CVD=cerebrovascular disease. LATE-NC=limbic predominant age-related TDP-43 encephalopathy neuropathologic change. MSD=meso scale discovery. pT=phosphorylated threonine for immunohistochemical measures of tau. p-tau=phosphorylated tau for plasma levels.

**Table S6: Multivariable linear regression models investigating neuropathologic and plasma p-tau predictors of cognitive scores.**

| **Cognitive test** | **Outcome** | **Adj R^2^** | **Predictor** | **β-coefficient** | **LowerCL** | **UpperCL** | **p-value** |
| --- | --- | --- | --- | --- | --- | --- | --- |
| **CDR Sum of Boxes** | CDR Sum of Boxes | 0.25 | MSD p-tau181 | 0.1079 | -1.0906 | 1.3065 | 0.8551 |
|  |  |  | Braak stage | 1.0912 | 0.0243 | 2.1581 | 0.0453 |
|  |  |  | Thal phase | 0.4581 | -0.4722 | 1.3884 | 0.3222 |
|  |  |  | Plasma to death | 1.3458 | -0.6277 | 3.3193 | 0.1737 |
|  | CDR Sum of Boxes | 0.27 | MSD p-tau217 | 3.9035 | -4.4259 | 12.233 | 0.3457 |
|  |  |  | Braak stage | 0.8842 | -0.2347 | 2.0031 | 0.1169 |
|  |  |  | Thal phase | 0.1806 | -0.8857 | 1.2469 | 0.7315 |
|  |  |  | Plasma to death | 1.3052 | -0.6370 | 3.2474 | 0.1798 |
| **MMSE** | MMSE | 0.30 | MSD p-tau181 | -0.0877 | -0.7665 | 0.5910 | 0.7926 |
|  |  |  | Braak stage | -0.7601 | -1.3537 | -0.1665 | 0.0141 |
|  |  |  | Thal phase | -0.2814 | -0.8001 | 0.2373 | 0.2750 |
|  |  |  | Plasma to death | -0.0450 | -1.1919 | 1.1018 | 0.9363 |
|  | MMSE | 0.32 | MSD p-tau217 | -2.1001 | -7.2021 | 3.0019 | 0.4052 |
|  |  |  | Braak stage | -0.6666 | -1.2934 | -0.0397 | 0.0380 |
|  |  |  | Thal phase | -0.1638 | -0.7536 | 0.4259 | 0.5729 |
|  |  |  | Plasma to death | -0.0800 | -1.2102 | 1.0501 | 0.8854 |

Analyses were performed on n=34 individuals for CDR and n=31 for MMSE. Cognitive test date nearest to death was used to evaluate neuropathologic and plasma p-tau predictors of variability in CDR and MMSE. Although time from plasma draw date to death (measured in years) was not observed to associate with plasma p-tau levels, this time factor was included in the model to adjust for any differences in time lag. Case with high creatinine was not included. All variables in model are shown. Variance inflation factor for all terms were below 3, which fell below the general cutoff of 5 used to indicate multicollinearity. Acronyms: Adj=adjusted. CDR Sum of Boxes=clinical dementia rating sum of boxes. CL=95% confidence limit. Acronyms: MSD=meso scale discovery. MMSE=mini mental state examination. P-tau=phosphorylated tau for plasma levels.

**Table S7. Clinical diagnosis and availability of tau PET observed within each neuropathologic grouping.**

|  | **Neuropathologic grouping** | | | | |
| --- | --- | --- | --- | --- | --- |
|  | **PSP (n=2)** | **AD (n=9)** | **AGD (n=2*)** | **PART (n=14)** | **PA (n=7)** |
| **Clinical diagnosis** |  |  |  |  |  |
| Cognitively unimpaired, % | 1/2 (50%) | 2/9 (22%) | 1/2 (50%) | 14/14 (100%) | 6/7 (86%) |
| Mild cognitive impairment | 0/2 (0%) | 2/9 (22%) | 1/2 (50%) | 0/14 (0%) | 1/7 (14%) |
| AD dementia | 0/2 (0%) | 3/9 (33%) | 0/2 (0%) | 0/14 (0%) | 0/7 (0%) |
| Non-AD neurodegenerative disorder |  |  |  |  |  |
| Dementia hard to classify | 0/2 (0%) | 2/9 (22%) | 0/2 (0%) | 0/14 (0%) | 0/7 (0%) |
| Progressive supranuclear palsy | 1/2 (50%) | 0/9 (0%) | 0/2 (0%) | 0/14 (0%) | 0/7 (0%) |
| **Tau PET availability** | 0/2 (0%) | 4/9 (44%) | 0/2 (0%) | 5/14 (36%) | 1/7 (14%) |

*AGD case with high creatinine was not included in table, as the cognitively unimpaired individual was excluded from clinicopathologic analyses after plasma creatinine levels were found to be an outlier influencing plasma p-tau levels. Note: The clinical diagnoses for AD neuropathologic grouping does not sum to 100% due to rounding rules. Acronyms: AD=Alzheimer’s disease. AGD=Argyrophilic grains disease. PA=pathological aging. PART=primary age-related tauopathy. PET=positron emission tomography. PSP=progressive supranuclear palsy.

# Supplemental Figures

**Figure S1. Immunohistochemical staining from hippocampus across disease groups.**

We commonly observed pT181 **(A-C,D-F)** and pT217 **(G-I,J-L)** immunostaining in tangle-bearing neurons and neuropil threads in the hippocampus of Alzheimer’s disease (84 y.o. male, Braak VI, Thal 5) **(A, G)** pathological aging (88 y.o. male, Braak II, Thal 3) **(B, H)** primary age related tauopathy (90 y.o. male, Braak III, Thal 1) **(C, I)**, argyrophilic grain’s disease (89 y.o. female, Braak II, Thal 0) (**D, J**; grains inset), and progressive supranuclear palsy (79 – 84 y.o. male, Braak III, Thal 3) **(E, K)**. Nonpathologic tau immunostaining of axons (83 y.o. female, Braak III, Thal 0) was observed in some case on pT181 **(F)**, but not in pT217 **(L)**. Scale bar represents 100 µm. Acronyms: AD=Alzheimer’s disease. AGD=argyrophilic grain disease. PA=pathological aging. PART=primary age related tauopathy. PSP=progressive supranuclear palsy. P-tau=phosphorylated tau for plasma levels.


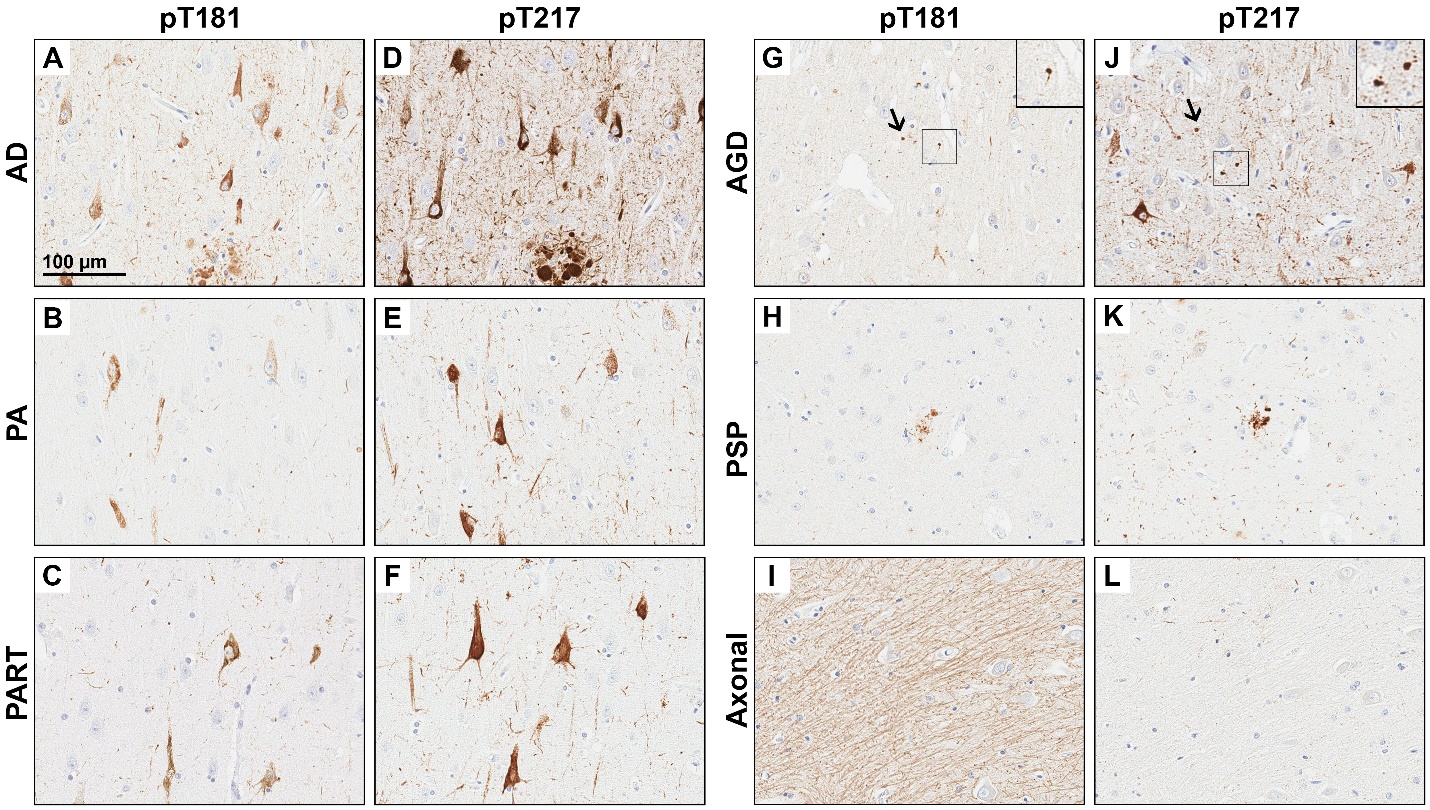


**Figure S2. The rostral-to-caudal neuroanatomic extent of the locus coeruleus.**

The rostral locus coeruleus (LC) was neuroanatomically assigned based upon constrained superior cerebellar peduncle (SCP), presence of cerebral aqueduct and the absence of the 4^th^ ventricle and the velum. The middle LC was assigned based upon an SCP that was observed to emerge caudally, as well as the presence of the 4^th^ ventricle and the velum. The caudal LC was assigned when the SCP fully emerged caudally, presence of the 4^th^ ventricle, and absence of the velum. Neuroanatomic interpretation of LC levels utilized recommendations from German et al.[3] Acronyms: CA=cerebral aqueduct. LC= locus coeruleus. SCP=superior cerebellar peduncle.


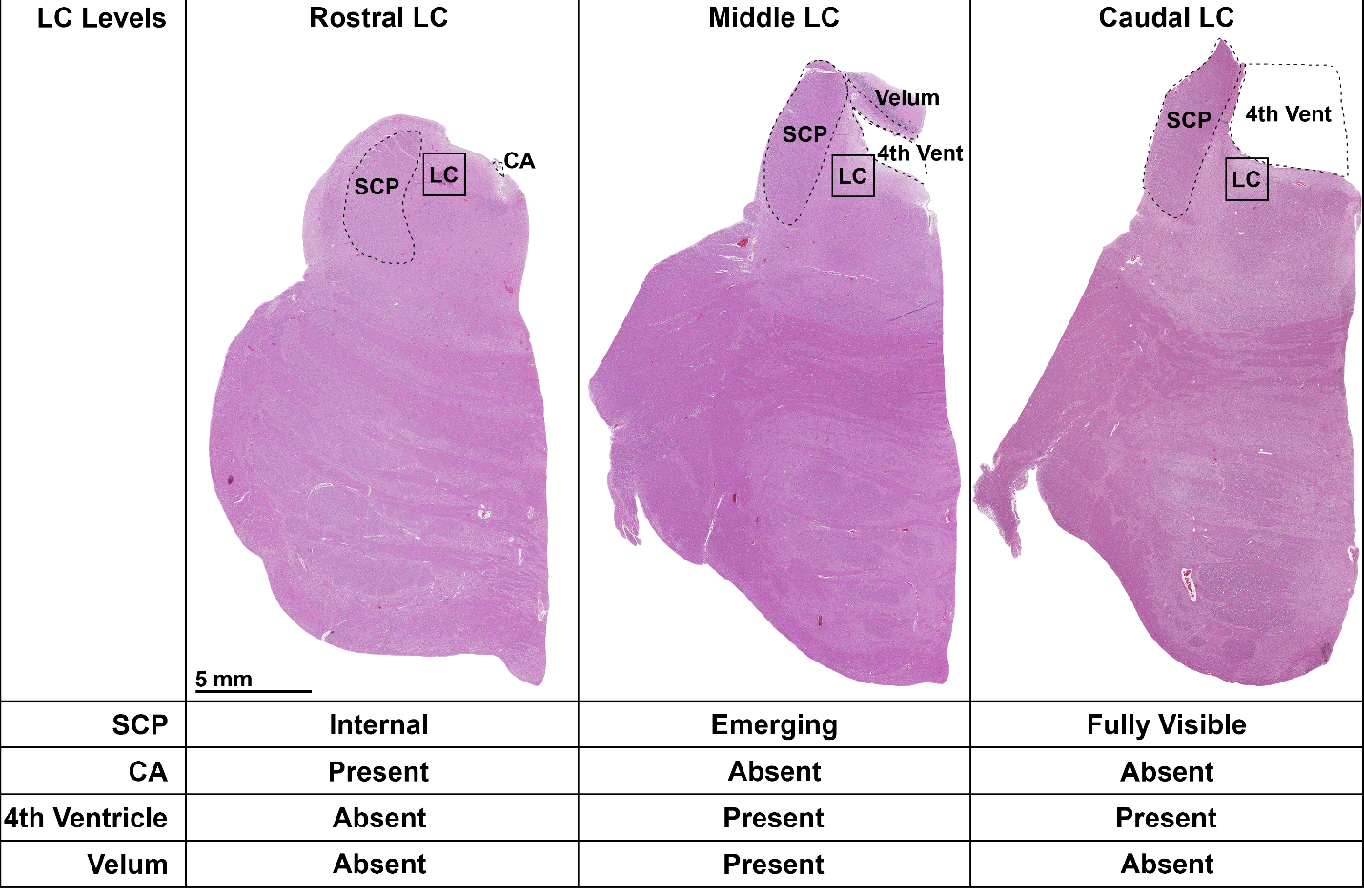


**Figure S3. The anterior-to-posterior neuroanatomic extent of the nucleus basalis of Meynert.**

The pre-anterior nucleus basalis of Meynert (nbM) was neuroanatomically assigned based upon presence of the nucleus accumbens and the absence of both the anterior commissure (AC) and globus pallidus (GP). The anterior nbM was assigned based on an AC that was observed decussating, split, or ventral to putamen, a GP that was either full or split, and either the hypothalamus or fornix presence. The intermediate nbM was assigned based upon the AC observed descending into temporal stem, a split GP, and presence of the mamillary body present. Neuroanatomic interpretation of nbM levels utilized recommendations previously described in detail [5, 7]. Acronyms: AC=anterior commissure. GP=globus pallidus. Hypothal=hypothalamus. MB=mamillary body. NA=nucleus accumbens. nbM=nucleus basalis of Meynert. O=optic.


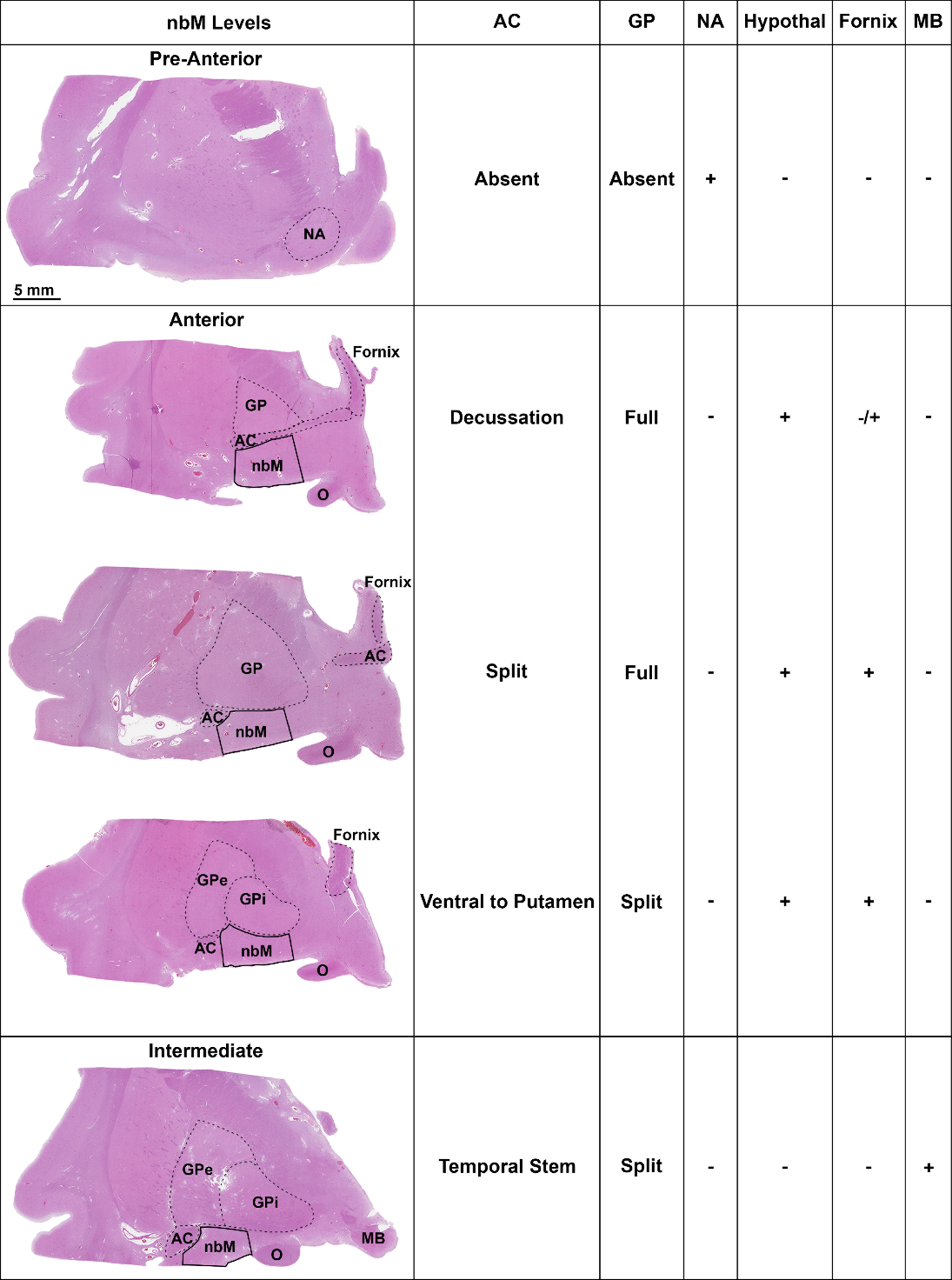


**Figure S4. Digital pathology analysis of locus coeruleus and nucleus basalis of Meynert.**

Low magnification image of H&E-stained pons tissue section with 1800x1800 µm^2^ ROI in green **(A)**. A high magnification of the LC showing neuromelanin containing neurons **(B)**. The custom-designed neuronal count markup displays counted neurons in orange. Regions in gray were recognized to be neither LC neurons or neuropil by the pattern recognition software and were thus excluded from the neuronal count macro to enhance accuracy **(C)**. Low magnification image of the H&E-stained nbM tissue section with manually annotated ROI in green **(D)**. A high magnification of the nbM showing the neurons of interest. The neuronal count markup displays counted neurons in orange. Regions in gray were recognized by the pattern recognition software to be neither nbM neurons or neuropil and were thus excluded from the neuronal count macro **(F)**. Scale bar represents 5 mm in 1x view **(A,D)** and 100 µm **(B,C,E,F)**. Acronyms: LC=locus coeruleus. H&E=hematoxylin and eosin. nbM=nucleus basalis of Meynert. ROI=region of interest.


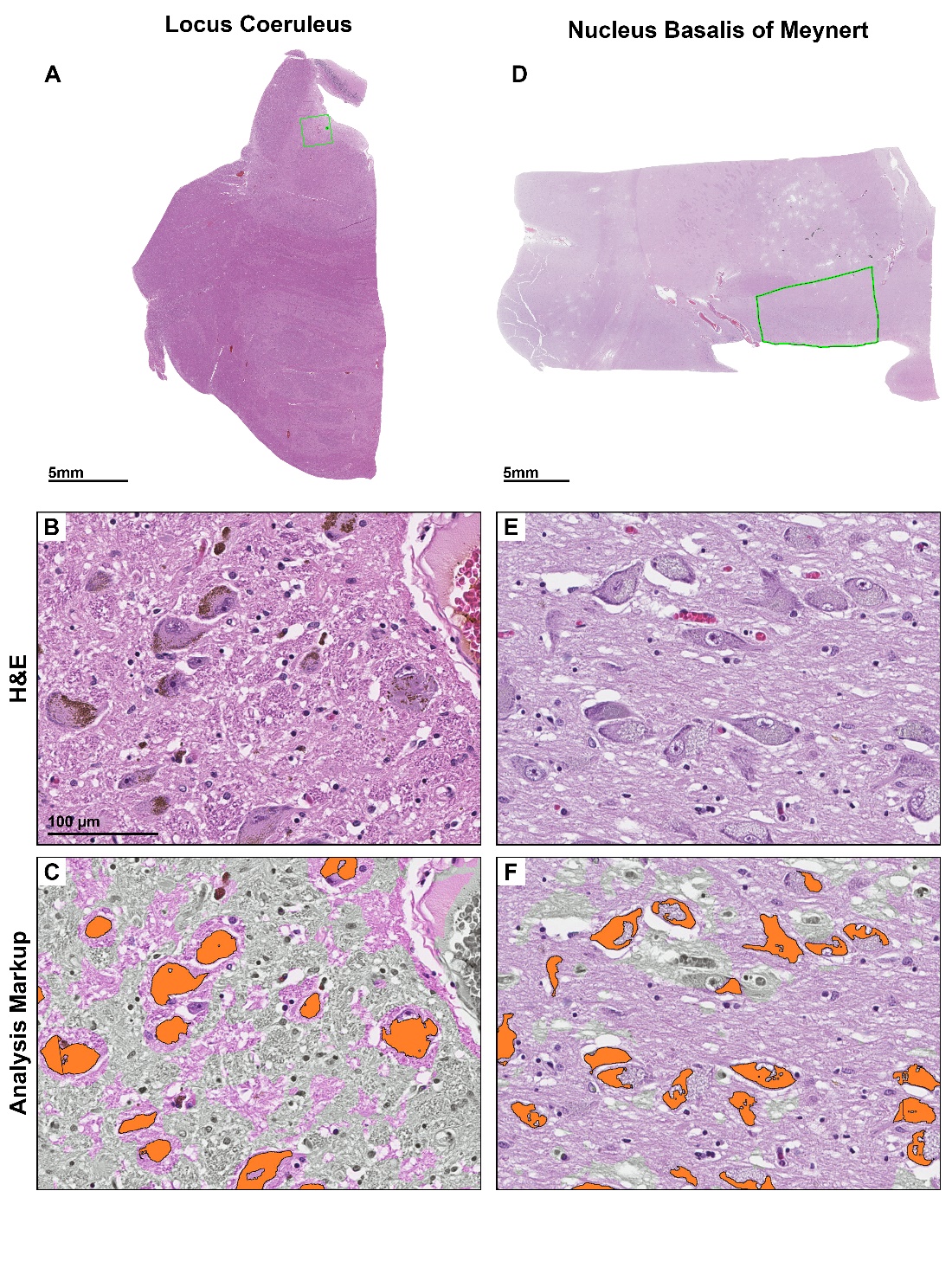


**Figure S5.** **Evaluation of antemortem contributors to plasma p-tau variability.**

Plasma p-tau 181 **(A-E)** and plasma p-tau217 **(F-J)** was investigated for outliers contributing to variability as described in Supplemental Results (p 2). When examining serum creatinine levels from the kidney, we observed an outlier influencing the relationship with plasma p-tau levels. This outlier is displayed in subsequent graphs and included in Spearman correlations in this figure, but was removed from all subsequent analyses **(A, F)**. Levels of serum aspartate aminotransferase in the liver **(B, G)** and alanine aminotransferase in the liver **(C, H)** did not associate with plasma p-tau. Neither age at plasma p-tau draw **(D, I)** or time from plasma p-tau draw to death **(E, J)** associated with plasma p-tau levels. Spearman correlation and corresponding significance is shown. Trendline with 95% confidence interval was computed from a linear model. ALT=alanine aminotransferase. AST=aspartate aminotransferase. mg/dL=milligrams per deciliter. Acronyms: MSD=meso scale discovery. PA=pathological aging. PART=primary age-related tauopathy. pg/mL=picograms per milliliter. PSP=progressive supranuclear palsy. p-tau=phosphorylated tau for plasma levels. U/L= units per liter. yrs=years.


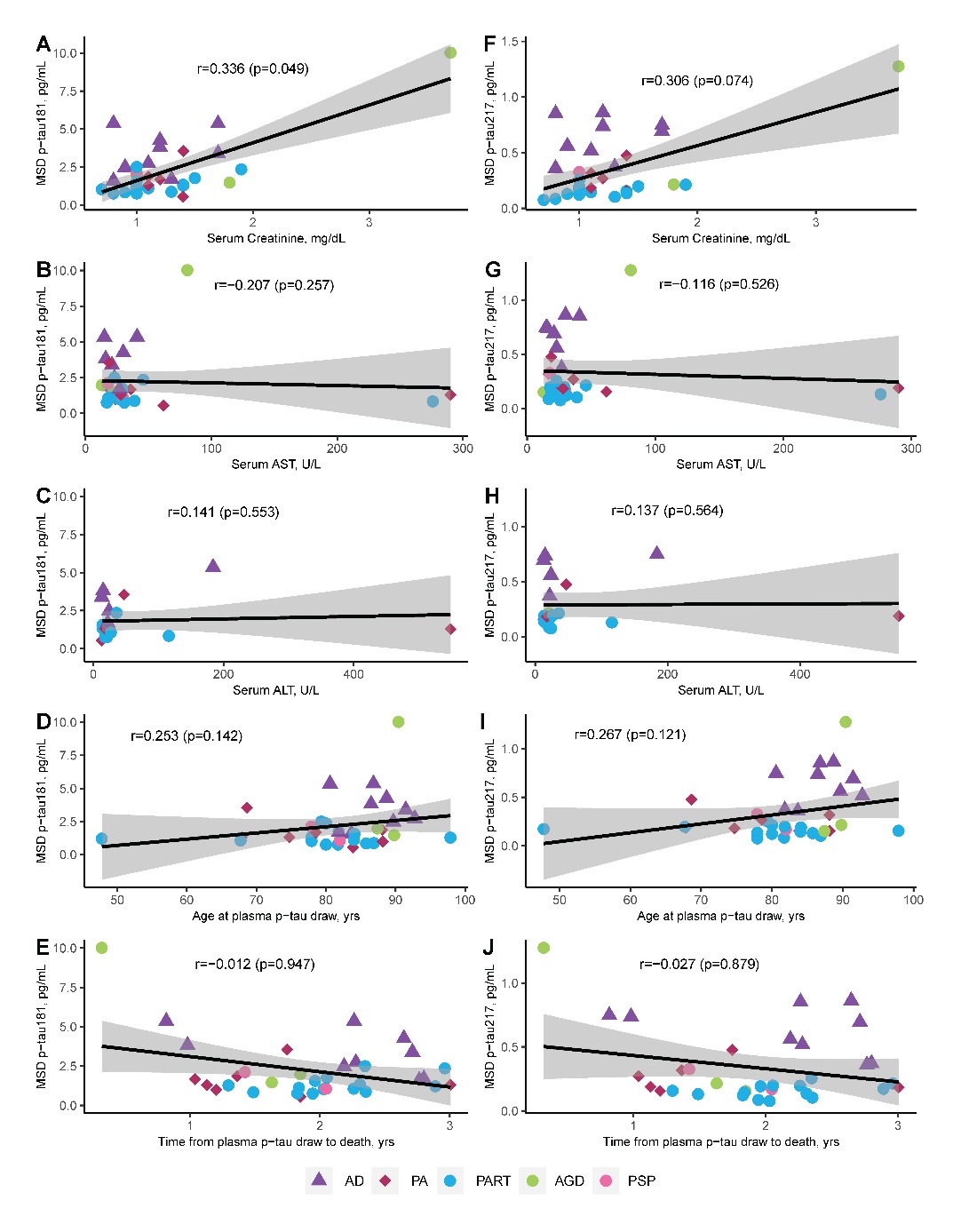


**Figure S6. Neuropathologic evaluation of regional digital pathology measures of tau and amyloid-β pathology in comparison to plasma p-tau181 and p-tau217 in CA1-subiculum subsectors of hippocampus.**

Utilizing the same epitope to immunohistochemically evaluate regional tau pathology in hippocampus, tau burden measures were compared to p-tau plasma levels **(A, C)**. pT181 tau burden measures approached significance with p-tau181 plasma levels **(A)**, with association even not observed between pT217 and p-tau217 plasma levels **(C)**. Digital pathology measures of amyloid-β (6F/3D) were additionally compared to plasma p-tau levels **(B, D)**. Amyloid-β burden associated with ptau-181 **(B)**. The strongest overall association of digital pathology measures in hippocampus was observed between amyloid-β (6F/3D) and p-tau217 **(D)**. P-tau181 **(A-B)** and p-tau217 **(C-D)** were examined across all individuals studied with AD shown as triangles, PA as diamonds, and primary tauopathies as circles. Spearman correlation and corresponding significance is shown. Case with high creatinine was not included. Trendline with 95% confidence interval was computed from a linear model. Acronyms: AD=Alzheimer’s disease. AGD=argyrophilic grains disease. MSD=meso scale discovery. PA=pathological aging. PART=primary age-related tauopathy. PSP=progressive supranuclear palsy. pT=phosphorylated threonine for immunohistochemical measures of tau. p-Tau=phosphorylated tau for plasma levels.

**
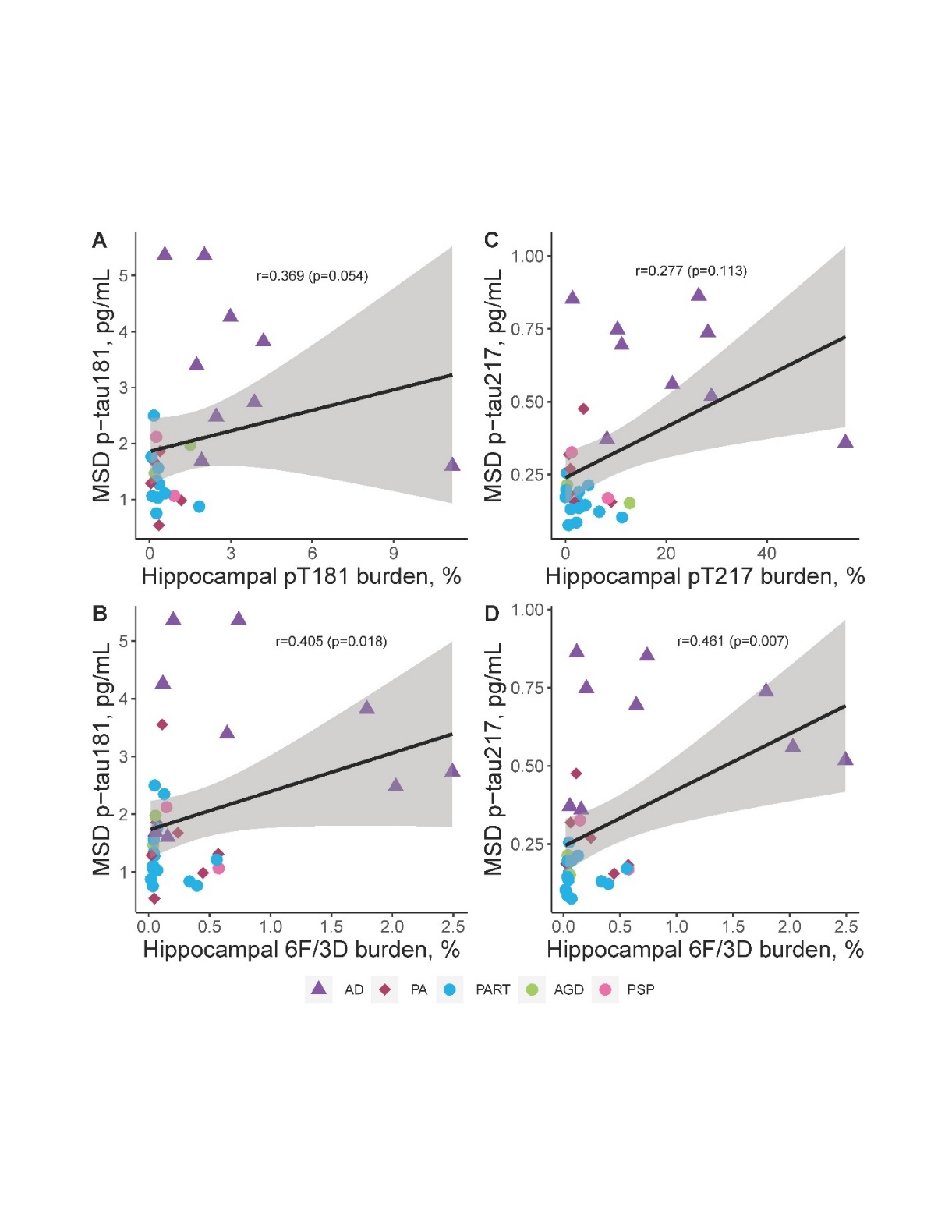
**

**Figure S7. Neuropathologic evaluation of regional digital pathology measures of neurotransmitter hub neuron count in comparison to plasma p-tau181 and p-tau217 in nucleus basalis of Meynert and locus coeruleus.** The nbM neuronal count did not associate with either p-tau181 plasma levels **(A)** or p-tau217 plasma levels **(C)**. Digital pathology measures of amyloid-β (6F/3D) were additionally compared to plasma p-tau levels **(B, D)**. However, lower LC neuronal count was observed to strongly associate with higher p-tau181 plasma levels **(B)** and higher p-tau217 plasma levels **(D)**. P-tau181 **(A-B)** and p-tau217 **(C-D)** were examined across all individuals studied with AD shown as triangles, PA as diamonds, and primary tauopathies as circles. Spearman correlation and corresponding significance is shown. Case with high creatinine was not included. Trendline with 95% confidence interval was computed from a linear model. Acronyms: AD=Alzheimer’s disease. AGD=argyrophilic grains disease. MSD=meso scale discovery. PA=pathological aging. PART=primary age-related tauopathy. PSP=progressive supranuclear palsy. pT=phosphorylated threonine for immunohistochemical measures of tau. p-Tau=phosphorylated tau for plasma levels.

**
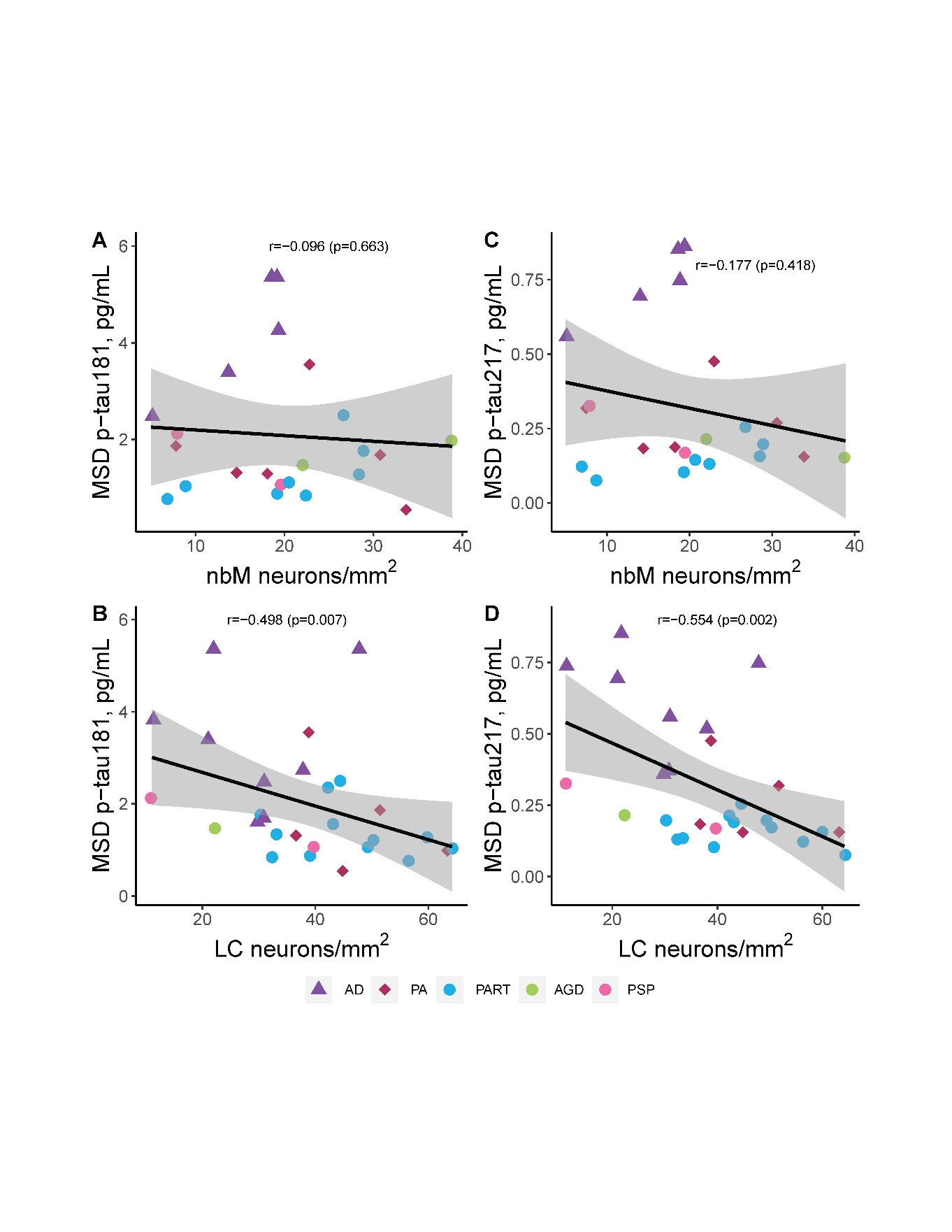
**

**Figure S8. Evaluation of [^18^F]flortaucipir uptake in inferior parietal cortex and plasma p-tau levels.**

The relationship between regional [^18^F]flortaucipir uptake in inferior parietal cortex was investigated to extend the findings from immunohistochemical studies. We did not observe a relationship with plasma p-tau181 levels **(A)**, but did observe a strong relationship with plasma p-tau 217 **(B)**. Spearman correlation and corresponding significance is shown. Case with high creatinine was not included. Trendline with 95% confidence interval was computed from a linear model. Acronyms: AD=Alzheimer’s disease. AGD=argyrophilic grains disease. MSD=meso scale discovery. PA=pathological aging. PART=primary age-related tauopathy. PET=positron emission tomography. PSP=progressive supranuclear palsy. pT=phosphorylated threonine for immunohistochemical measures of tau. p-Tau=phosphorylated tau for plasma levels. SUVR=standard uptake value ratio.


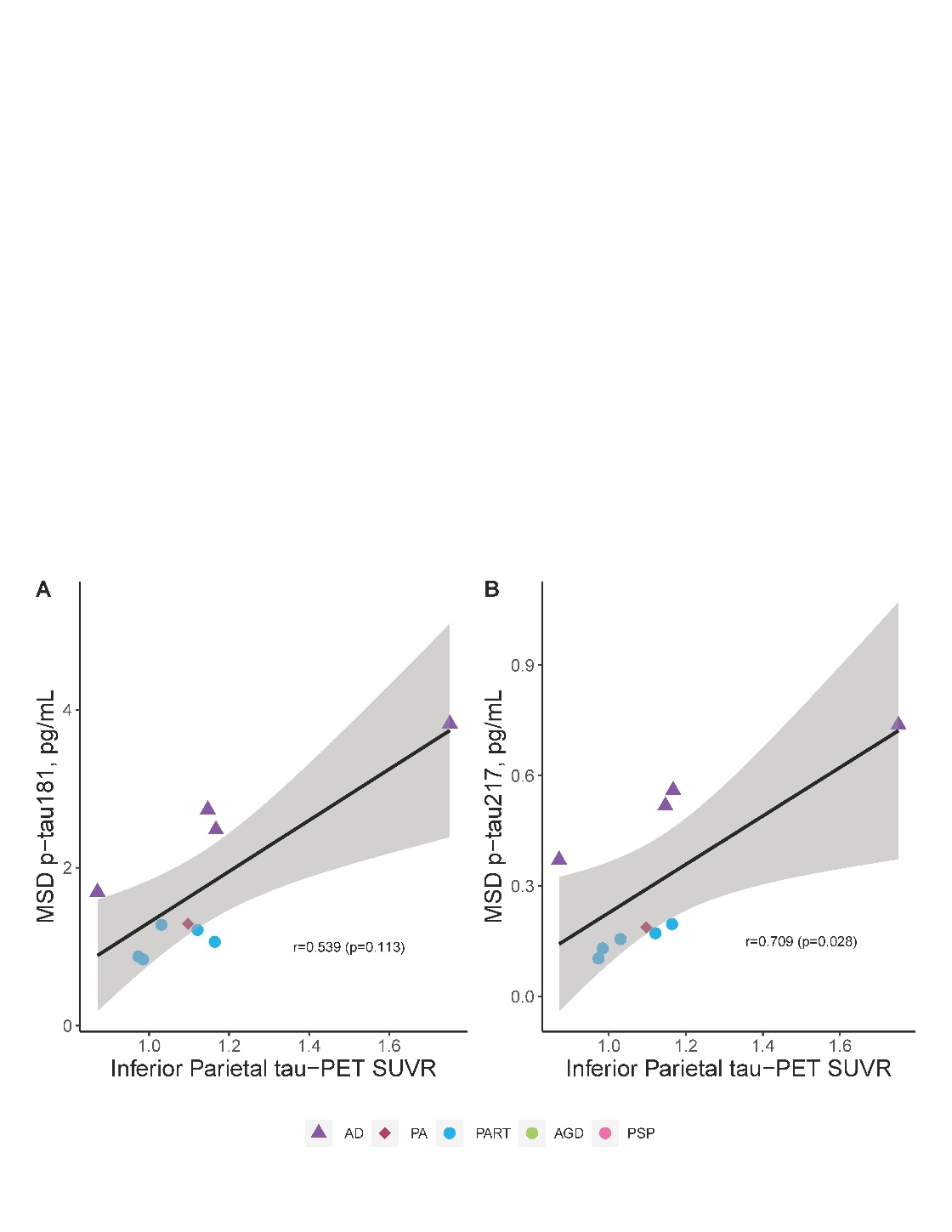


**Figure S9. Predictive modeling of Alzheimer’s disease neuropathologic change.**

Predictive modeling of neuropathologic diagnosis of intermediate-to-high Alzheimer’s disease neuropathologic change compared to none-to-low show highest Area Under the Curve for p-tau217, followed by clinical dementia rating sum of boxes, p-tau181, and mini mental state examination. Case with high creatinine was not included. Given the sample size and use of logistic regression, plasma p-tau levels and cognitive scores were modeled individually, and were covariates not included. Acronyms: AUC=Area Under the Curve. CDRsum=clinical dementia rating sum of boxes. MMSE=mini mental state examination.


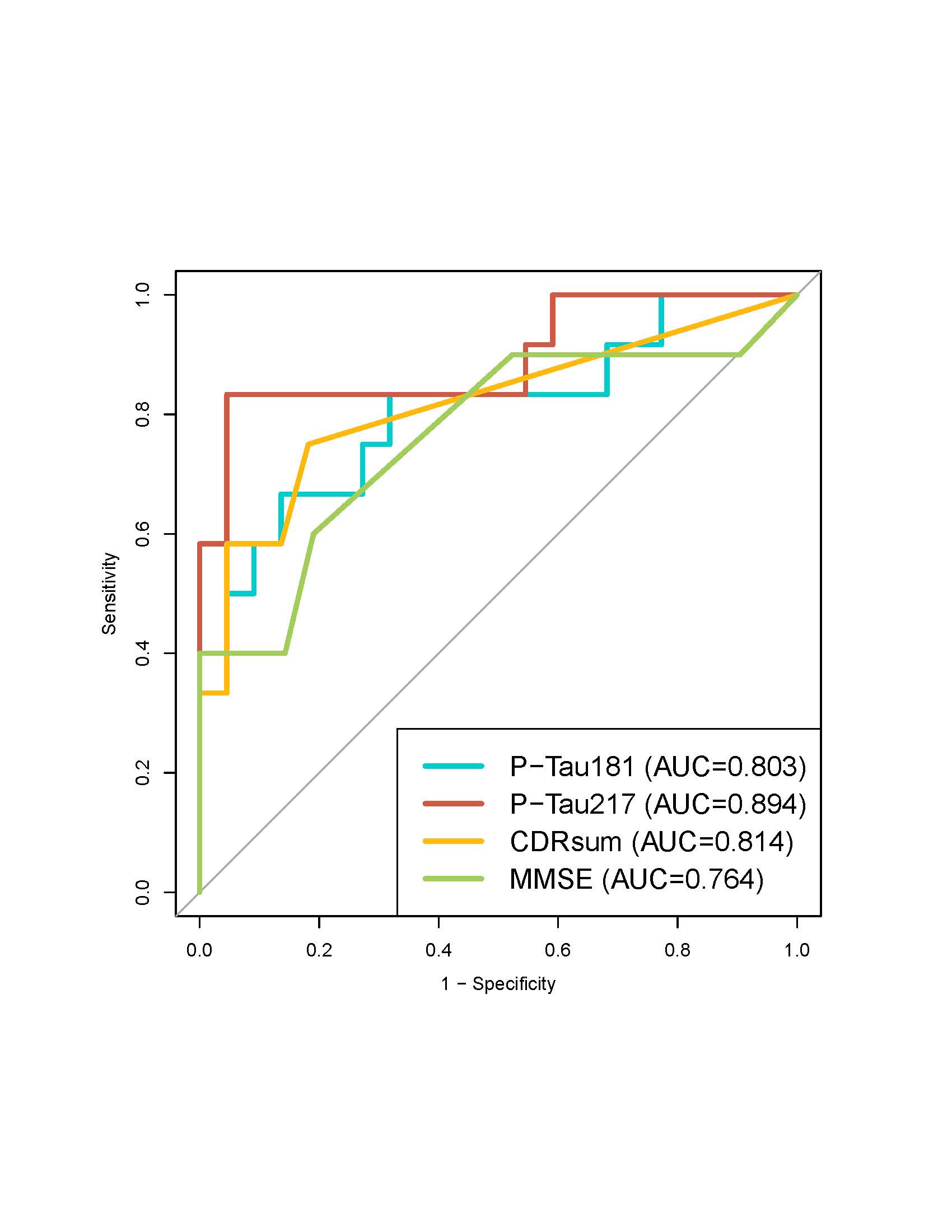


# eReferences

1. Mielke MM, Frank RD, Dage JL, Jeromin A, Ashton NJ, Blennow K, Karikari TK, Vanmechelen E, Zetterberg H, Algeciras-Schimnich A, et al: **Comparison of Plasma Phosphorylated Tau Species With Amyloid and Tau Positron Emission Tomography, Neurodegeneration, Vascular Pathology, and Cognitive Outcomes.** *JAMA Neurol* 2021, **78:**1108-1117.

2. Murray ME, Graff-Radford NR, Ross OA, Petersen RC, Duara R, Dickson DW: **Neuropathologically defined subtypes of Alzheimer's disease with distinct clinical characteristics: a retrospective study.** *Lancet neurology* 2011, **10:**785-796.

3. German DC, Walker BS, Manaye K, Smith WK, Woodward DJ, North AJ: **The human locus coeruleus: computer reconstruction of cellular distribution.** *J Neurosci* 1988, **8:**1776-1788.

4. Theofilas P, Ehrenberg AJ, Dunlop S, Di Lorenzo Alho AT, Nguy A, Leite REP, Rodriguez RD, Mejia MB, Suemoto CK, Ferretti-Rebustini REL, et al: **Locus coeruleus volume and cell population changes during Alzheimer's disease progression: A stereological study in human postmortem brains with potential implication for early-stage biomarker discovery.** *Alzheimers Dement* 2017, **13:**236-246.

5. Hanna Al-Shaikh FS, Duara R, Crook JE, Lesser ER, Schaeverbeke J, Hinkle KM, Ross OA, Ertekin-Taner N, Pedraza O, Dickson DW, et al: **Selective Vulnerability of the Nucleus Basalis of Meynert Among Neuropathologic Subtypes of Alzheimer Disease.** *JAMA Neurol* 2019.

6. Mesulam M, Shaw P, Mash D, Weintraub S: **Cholinergic nucleus basalis tauopathy emerges early in the aging-MCI-AD continuum.** *Ann Neurol* 2004, **55:**815-828.

7. Mesulam MM, Geula C: **Nucleus basalis (Ch4) and cortical cholinergic innervation in the human brain: observations based on the distribution of acetylcholinesterase and choline acetyltransferase.** *J Comp Neurol* 1988, **275:**216-240.

8. Lowe VJ, Wiste HJ, Senjem ML, Weigand SD, Therneau TM, Boeve BF, Josephs KA, Fang P, Pandey MK, Murray ME, et al: **Widespread brain tau and its association with ageing, Braak stage and Alzheimer's dementia.** *Brain* 2018, **141:**271-287.

9. Mielke MM, J.L. D, R.D. F, A. A-S, D.S. K, V.J. L, G. B, P. V, J. G-R, C.R. JJ, R.C. P: **Performance of plasma phosphorylated tau 181 and 217 in the community.** *Nature Medicine* 2022, **In Press**.

10. Nho K, Kueider-Paisley A, Ahmad S, MahmoudianDehkordi S, Arnold M, Risacher SL, Louie G, Blach C, Baillie R, Han X, et al: **Association of Altered Liver Enzymes With Alzheimer Disease Diagnosis, Cognition, Neuroimaging Measures, and Cerebrospinal Fluid Biomarkers.** *JAMA Netw Open* 2019, **2:**e197978.
